# Pembrolizumab plus platinum-based chemotherapy for unfavorable cancer of unknown primary site: Case report

**DOI:** 10.1016/j.amsu.2020.10.024

**Published:** 2020-10-17

**Authors:** Teppei Kamada, Hiroshi Ishiguro, Shinya Okada, Hideyuki Takeuchi, Junji Takahashi, Keigo Nakashima, Yuichi Nakaseko, Norihiko Suzuki, Hironori Ohdaira, Yutaka Suzuki

**Affiliations:** a Department of Surgery, International University of Health and Welfare Hospital, 537-3, Iguchi, Nasushiobara, Tochigi, 329-2763, Japan; b Department of Oncology, International University of Health and Welfare Narita Hospital, 852, Hatakeda, Narita, Chiba, 286-0124, Japan; c Department of Pathology, International University of Health and Welfare Hospital, 537-3, Iguchi, Nasushiobara, Tochigi, 329-2763, Japan

**Keywords:** Cancer of unknown primary, Non-small-cell lung cancer, Pembrolizumab, Pemetrexed, Platinum, CUP, cancer of unknown primary site, NCCN, National Comprehensive Cancer Network, PD-L1, programmed death ligand 1, EGFR, epidermal growth factor receptor, ALK, anaplastic lymphoma kinase, CR, complete response, LDH, lactate dehydrogenase, CEA, carcinoembryonic antigen, CT, computed tomography, PET, positron emission tomography, CK7, cytokeratin 7, TTF-1, thyroid transcription factor-1

## Abstract

**Introduction:**

We report a case of sustained complete response in unfavorable cancer of unknown primary site (CUP) successfully treated with chemotherapy combining pembrolizumab, pemetrexed and platinum.

**Case presentation:**

A 66-year-old man was presented with weight loss and cough for 3 months. Contrast-enhanced computed tomography (CT) confirmed a mass in the superior anterior mediastinum and multiple enlarged mediastinal and axillary lymph nodes. Positron emission tomography-CT (PET-CT) showed abnormal uptake in the corresponding lesions. Histopathological analysis of the left axillary nodule revealed poorly differentiated adenocarcinoma. Immunohistochemistry showed the tumor cells were positive for cytokeratin 7 and thyroid transcription factor-1 and negative for cytokeratin 20. Thus, the patient was diagnosed as poorly differentiated adenocarcinoma of unknown primary, and treated as non-small-cell lung cancer. Additional genetic testing revealed the patient was negative for EGFR, ALK fluorescence in situ hybridization, ROS1, BRAF, and PD-L1 22C3 IHC with Tumor Proportion Score (TPS) was less than 1%. The patient received six cycles of pembrolizumab, platinum, and pemetrexed intravenously. Cisplatin was switched to carboplatin because of cisplatin nephrotoxicity in one course. PET-CT after six cycles showed all lesions disappeared; complete response was considered to have been achieved. Maintenance therapy of pembrolizumab and pemetrexed has been continued for 6 months after the induction therapies to prevent progressive disease. Complete response has been maintained.

**Discussion:**

Chemotherapy with pembrolizumab, platinum and pemetrexed could be valuable for treating unfavorable CUP.

**Conclusion:**

Chemotherapy with pembrolizumab, platinum, and pemetrexed helped achieved sustained complete response in a patient with unfavorable CUP.

## Highlights

• The prognosis of patients with unfavorable CUP remains poor.
• Pembrolizumab's addition to standard chemotherapy resulted in significantly longer overall survival for patients with NSCLC.
• Chemotherapy with pembrolizumab, platinum, and pemetrexed helped achieved sustained complete response in a patient with unfavorable CUP.

## 1 Introduction

Cancers of unknown primary site (CUP) represent a heterogeneous group of metastatic tumors for which a standardized diagnostic work-up fails to identify the origin site at diagnosis [1]. Treatments have been established for CUP with good prognoses and long-term survival can be expected. In contrast, the prognosis of patients with unfavorable CUP remains poor. Despite the recommendation of first-line combination regimens, including platinum- or taxane-based chemotherapy, the median overall survival was 9 months (95% CI: 8.1–9.8) and 1-year survival rate was 35.6% (95% CI: 32.0–39.3) [1,2]. According to the National Comprehensive Cancer Network (NCCN) guidelines for CUP, localized adenocarcinoma occurring in the mediastinum most likely derives from either a germ cell tumor or a non-small cell lung cancer (NSCLC). Patients aged 50 years or older should be treated per NCCN guidelines for non-small-cell lung cancer [3]. Among patients having NSCLC with a tumor proportion score of ≥50% for programmed death ligand 1 (PD-L1), pembrolizumab has replaced cytotoxic chemotherapy as the first-line treatment of choice [4]. In the KEYNOTE-189 trial, pembrolizumab's addition to standard chemotherapy comprising pemetrexed and a platinum-based drug resulted in significantly longer overall survival and progression-free survival than those associated with chemotherapy alone among patients with previously untreated metastatic nonsquamous NSCLC without epidermal growth factor receptor (EGFR) or anaplastic lymphoma kinase (ALK) mutations (ClinicalTrials.gov number, NCT02578680) [5]. We describe a case of unfavorable CUP successfully treated with pembrolizumab plus pemetrexed and platinum-based drug chemotherapy in which complete response (CR) has been maintained. The work has been reported in line with the SCARE criteria [6].

## 2 Presentation of case

A 66-year-old man was hospitalized due to weight loss (−13 kg) and cough for 3 months. He had no significant medical history but had a smoking history of 24 × 30 years. He is a cook and his hobby is golfing.

Blood examination on admission revealed the following results: Albumin, 3.8 g/dl; lactate dehydrogenase (LDH), 254 IU/L; blood urea nitrogen, 7.7 mg/dl; creatinine, 0.95 mg/dl; B-type natriuretic peptide, 20.5 pg/mL; HbA1c, 5.6%; C-reactive protein, 0.05 mg/dl; white blood cell count, 3660/ μL; hemoglobin, 13.5 g/dL; platelet count, 22.1 × 10^4^/ μL; carcinoembryonic antigen (CEA), 2761 ng/ml; and soluble interleukin 2 receptor, 501 U/mL. All other tumor markers were normal.

**Fig. 1:**
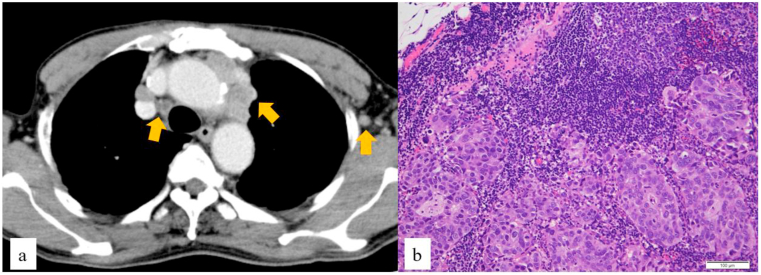
(a) Contrast-enhanced computed tomography showing a mass with an irregular margin and heterogenous enhancement (arrow) in the superior anterior mediastinum and multiple enlarged mediastinal and axillary lymph nodes (arrows). (b) Histopathological analysis of the left axillary nodule showing metastasis of poorly differentiated adenocarcinoma, which shows epithelial binding, glandular cavity formation, and light cytoplasm (hematoxylin and eosin staining,×100 magnification).

**Fig. 2:**
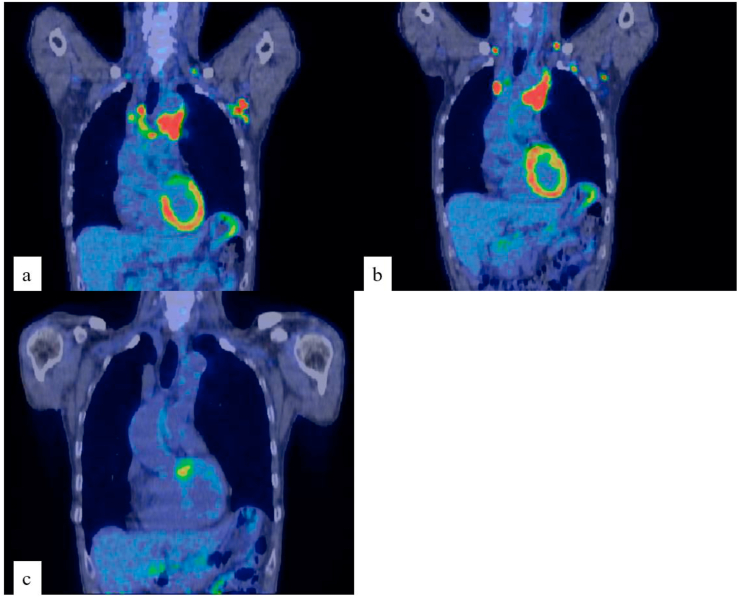
(a) (b) PET-CT showing abnormal uptake in bilateral subclavian, left axillary, mediastinum, and hilar lymph nodes, and in a mass in the superior anterior mediastinal area. (c) PET-CT showing disappearance of all lesions after six courses of chemotherapy. PET-CT: Positron emission tomography-computed tomography.

Electrocardiography showed normal sinus rhythm. The ejection fraction was 51%. Echocardiography revealed a diffuse reduction in left ventricular wall movement. The Eastern Cooperative Oncology Group performance status score was 0. Contrast-enhanced computed tomography (CT) confirmed a mass with an irregular margin and heterogeneous enhancement in the superior anterior mediastinum and multiple enlarged mediastinal and axillary lymph nodes (Fig. 1a). Positron emission tomography-CT (PET-CT) showed abnormal uptake in bilateral subclavian, left axillary, mediastinal, and hilar lymph nodes and in a mass in the superior anterior mediastinum (Fig. 2a and b). Upper gastrointestinal endoscopy and colonoscopy were negative.

**Fig. 3:**
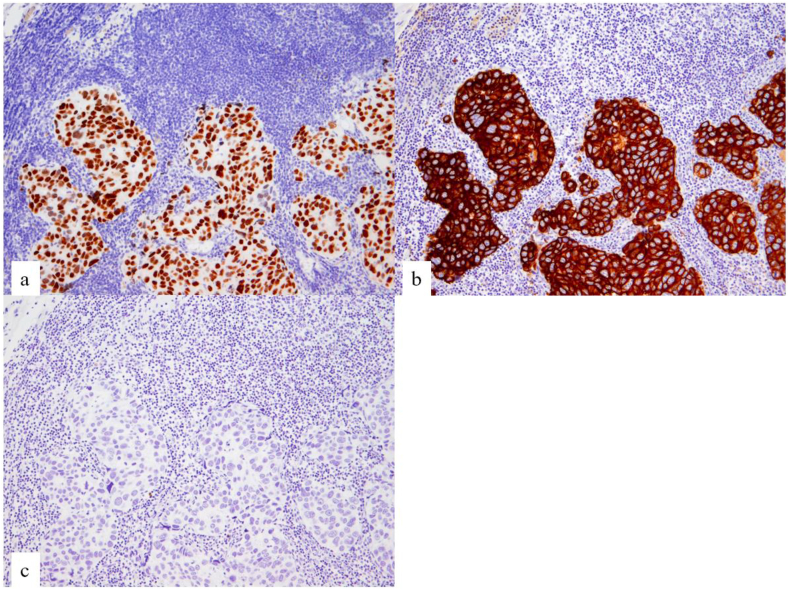
Immunohistochemical staining. (a) Tumor cells positive for thyroid transcription factor-1. (b) Tumor cells positive for cytokeratin 7. (c) Tumor cells negative for cytokeratin 20.

CUP with multiple lymph node metastases was diagnosed. Histopathological examination of the left axillary nodule confirmed metastasis of poorly differentiated adenocarcinoma, which showed epithelial binding, glandular cavity formation, and light cytoplasm (Fig. 1b). Immunohistochemistry showed the tumor cells were positive for cytokeratin 7 (CK7), CEA, CK (AE1/AE3, CAM5.2), and thyroid transcription factor-1 (TTF-1) and negative for CK20, estrogen receptor, progesterone receptor, CD5, CD10, neuron-specific enolase, P40, PAX8, GATA3, PSA, CDX2, and NapsinA (Fig. 3 a, b, c). Histopathological and immunohistochemical examinations suggested that the germ-cell tumor was unlikely. These results were in line with those associated with NSCLC.

The patient was treated as NSCLC according to the NCCN guidelines for CUP [3]: localized adenocarcinoma, mediastinal clinical presentation, TTF-1 positivity, and age of ≥50 years. Additional genetic testing revealed the patient was negative for ALK fluorescence in situ hybridization, ROS1, BRAF, and EGFR and PD-L1 22C3 IHC with Tumor Proportion Score (TPS) of <1%. Considering his job (cook) and hobby (golf), we selected a regimen that would not cause taxane-based neurotoxicity.

He was treated with pembrolizumab, cisplatin, and pemetrexed in line with the KEYNOTE-189 trial [5] for the NSCLC. However, cisplatin was switched to carboplatin because of severe cisplatin nephrotoxicity in one course. The patient received six cycles of 200 mg of pembrolizumab, 350 mg of carboplatin, and 800 mg of pemetrexed intravenously on day 1 every 3 weeks. Pancytopenia and fatigability were noted during chemotherapy; however, six courses of induction therapy were completed while resorting to drug withdrawal. PET-CT performed to evaluate the treatment response after six courses according to the Response Evaluation Criteria in Solid Tumors (RECIST) guidelines [7] showed disappearance of all lesions and CEA within the normal range (4.8 ng/mL); therefore, CR was considered to have been achieved (Fig. 2c). Maintenance therapy with 200 mg of pembrolizumab and 800 mg of pemetrexed was introduced after the induction therapies to prevent progressive disease. No recurrence has been observed for 12 months and achieved return to society.

## 3 Discussion

CUP are defined as histologically confirmed cancers manifesting in the advanced stage, with no identifiable primary site upon the use of standard diagnostic procedures. They account for 3–5% of all malignancies and have extremely poor prognoses [1,2]. CUP include a wide variety of cancer types, There are established treatments for a subtype associated with long term survival [1]. However, the median overall survival was 3–4 months in a cohort study that excluded a good prognosis group and these patients cannot receive effective treatment [8,9].

First-line chemotherapy with platinum- or taxane-based combinations is recommended for the poor prognosis group of CUP; however, the outcomes were unsatisfactory [1,2,[10], [11], [12], [13], [14]]. In our patient, we treated CUP as NSCLC due to adenocarcinoma localized to the mediastinum, TTF-1 positivity, and age higher than 50 years. Among patients with a tumor proportion score of ≥50% for PD-L1 and lacking sensitizing EGFR or ALK mutations, pembrolizumab has replaced cytotoxic chemotherapy as the first-line treatment of choice [4]. However, patients with a tumor proportion score of ≥50% represent a minority of those with NSCLC. For those with a tumor proportion score of <1%, pembrolizumab is recommended as the second-line treatment. However, patients with advanced NSCLC could deteriorate rapidly, and less than 50% of patients with advanced NSCLC receive second-line therapy [15,16].

In 2018, a double-blind, phase 3 trial in patients with previously untreated metastatic nonsquamous NSCLC without EGFR or ALK mutations, addition of pembrolizumab to standard chemotherapy with pemetrexed and a platinum-based drug, compared to chemotherapy alone, resulted in significantly longer overall survival and progression-free survival [5]. Modulation of immune responses through inhibitors of programmed death-1 may be enhanced by the potential immunogenic effects of cytotoxic chemotherapy, such as increasing the potential for antigen cross-presentation by dendritic cells after the destruction of tumor cells [17], inhibiting myeloid-derived suppressor cells [18], increasing the ratio of cytotoxic lymphocytes to regulatory T cells [19], and blocking the STAT6 pathway to enhance dendritic-cell activity [20]. In this trial, the response rate was higher in the pembrolizumab combination group than in the placebo-combination group across all categories of PD-L1 tumor proportion score (≥50 [*n* = 132], 1–49 [*n* = 128], <1 [*n* = 127], and could not be evaluated [*n* = 23]). However, differences in the response rate were found across categories (61.4% vs 48.4% vs 32.3%). The response rate was higher in the patients with a tumor proportion score of ≥50% for PD-L1 but was lower in the patients with a tumor proportion score of <1%. Furthermore, CR was confirmed in only two patients (0.48%) of 410 in the pembrolizumab combination group. In our case, the tumor proportion score was <1%; however, CR was confirmed in a short period.

We chose cisplatin at first; however, the patient showed extensive nephrotoxicity in the first course; therefore, we switched to carboplatin quickly. The regimen of pembrolizumab, pemetrexed, and carboplatin was chosen because this combination can be continuously used even in patients with low cardiac function and maintains quality of life. Thus, effective chemotherapy was ensured while the patient continued his job and hobby.

Seve et al. reported a worse prognosis in CUP patients with a performance status score of ≥2, high overall comorbidity score, liver metastasis, elevated serum LDH levels, lymphopenia (defined as an absolute lymphocyte count of ≥0.7 × 10^9^/ L), and low serum albumin levels [9]. CR was achieved in our patient since none of these factors was relevant in our case. Frequent adverse events have been reported for the pembrolizumab, carboplatin, and pemetrexed regimen. In the KEYNOTE-189 trial [5], adverse events of any cause and regardless of attribution to treatment occurred in 99.8% of the patients in the pembrolizumab combination group. These events were of grade 3 or higher in 67.2% of the patients. Pancytopenia and fatigability of grade 3 were observed in our patient. Drug withdrawal, transfusion, and granulocyte-colony stimulating factor, which effectively ensure continuous chemotherapy and relapse-free survival, were continued. Long-term follow-up will be necessary in the future. Effective chemotherapy is possible even for unfavorable CUP, for which few treatment options are available, by choosing the appropriate regimen for each patient by performing a quick and appropriate primary organ estimation. Chemotherapy with pembrolizumab, platinum, and pemetrexed can be valuable as a treatment option for unfavorable CUP.

## 4 Conclusion

Chemotherapy with pembrolizumab, platinum, and pemetrexed helped achieve CR in a patient with unfavorable CUP.

## Consent of patient

Written informed consent was obtained from the patient for publication of this case report and any accompanying images.

## Provenance and peer review

Not commissioned, externally peer reviewed.

## Source of funding

We have no sponsors.

## Ethical approval

This study has been exempted by our institution.

## Author contribution

TK: study design, data collection, data analysis, writing.

HI, SO, HO: critical revision.

YS: final approval of the article.

Any other authors: data collection.

All authors read and approved the final manuscript.

## Registration of research studies

This paper is case report. The authors don't need to register this work.

## Guarantor

Teppei Kamada, the corresponding author of this manuscript accept full responsibility for the work and the conduct of the study, access to the data and controlled the decision to publish.

## Declaration of competing interest

There are no conflicts of interest.
